# DNA methylation and gene expression in *Mimulus guttatus*

**DOI:** 10.1186/s12864-015-1668-0

**Published:** 2015-07-07

**Authors:** Jack M. Colicchio, Fumihito Miura, John K. Kelly, Takashi Ito, Lena C. Hileman

**Affiliations:** Department of Ecology and Evolutionary Biology, University of Kansas, Lawrence, KS 66045 USA; Department of Medical Biochemistry, Department of Biochemistry, Fukuoka 812-8581, Fukuoka 812-8582 Japan

## Abstract

**Background:**

The presence of methyl groups on cytosine nucleotides across an organism’s genome (methylation) is a major regulator of genome stability, crossing over, and gene regulation. The capacity for DNA methylation to be altered by environmental conditions, and potentially passed between generations, makes it a prime candidate for transgenerational epigenetic inheritance. Here we conduct the first analysis of the *Mimulus guttatus* methylome, with a focus on the relationship between DNA methylation and gene expression.

**Results:**

We present a whole genome methylome for the inbred line Iron Mountain 62 (IM62). DNA methylation varies across chromosomes, genomic regions, and genes. We develop a model that predicts gene expression based on DNA methylation (R^2^ = 0.2). *Post hoc* analysis of this model confirms prior relationships, and identifies novel relationships between methylation and gene expression. Additionally, we find that DNA methylation is significantly depleted near gene transcriptional start sites, which may explain the recently discovered elevated rate of recombination in these same regions.

**Conclusions:**

The establishment here of a reference methylome will be a useful resource for the continued advancement of *M. guttatus* as a model system. Using a model-based approach, we demonstrate that methylation patterns are an important predictor of variation in gene expression. This model provides a novel approach for differential methylation analysis that generates distinct and testable hypotheses regarding gene expression.

**Electronic supplementary material:**

The online version of this article (doi:10.1186/s12864-015-1668-0) contains supplementary material, which is available to authorized users.

## Background

DNA cytosine methylation is an epigenetic modification that acts in conjunction with histone modification and small RNAs to regulate gene expression [1–3] and control transposable elements [4, 5]. In addition, DNA methylation appears to alter mutation rates [6] and to decrease rates of recombination [7]. It is found in organisms spanning the eukaryotic phylogeny [8, 9], and can occur in many sequence contexts. In plants, cytosine methylation can be found in CG, CHG, or CHH contexts, where H is any nucleotide besides G [10]. It appears that much of the methylome is stable within an individual; however, the methylome does exhibit predictable plastic responses to developmental and environmental cues [11, 12].

Recent work has greatly expanded our knowledge of the mechanisms involved in maintaining and modifying DNA methylation in plants [13–18], yet we still do not fully understand how specific patterns of DNA methylation in and near coding sequences control gene expression. In *Arabidopsis thaliana*, CG DNA methylation in regulatory sequences is negatively correlated with gene expression [3, 19], possibly through limiting promoter accessibility. Contrastingly, gene body CG methylation is elevated in moderate to highly expressed genes [3, 10, 20], potentially though the removal of histone variant H2A.Z [21]. Similar patterns of association between the distribution of plant CG methylation and gene expression have been found in the wild rice [20], tomato [22], and maize [23]. Additionally, *Arabidopsis* genes within differentially methylated regions tended to be more highly expressed in individuals with increased CG methylation, but lower in individuals with increased non-CG (CHG and CHH) methylation [24]. However, the interaction between gene expression and different forms of DNA methylation in and around genes has not been fully explored. For example, the impact of non-CG methylation on gene expression is especially understudied, despite its established role in regulating transposable elements through pre- and post-transcriptional silencing [25].

The standard method for characterizing genomic patterns of DNA methylation is to classify genes into methylation quantiles and then compare gene expression across these groups [3, 20, 22, 26–29]. Here, we adopt an explicit model-based approach, predicting gene expression from gene methylation and other basic gene-specific features (exon length, intron length, and exon number). We compare the methylome of an inbred line, to gene expression from a distinct recombinant inbred line, and test how well DNA methylation, in combination with other stable genetic factors, predict gene expression across lines and tissue types. The explanatory power of stable epigenetic variation on gene expression is relatively unknown (although see [30] for model-based approaches to predicting gene expression via promoter motifs in *Saccharomyces cerevisiae,* and [31] for a Sanger sequencing approach to gene expression modeling based on histone and DNA methylation in rice). With the model-based approach presented here, we are able to assess the scale to which constitutive epigenetic variation effects global gene expression, and the patterns of DNA methylation through which this regulation is manifest.

Previous studies of *Mimulus guttatus* have demonstrated transgenerational epigenetic inheritance [32–35]. Herbivore induced defensive traits can be transmitted between generations, and the observed transcriptional basis of this response [11], has made it a promising model system in the burgeoning field of ecological epigenetics [36–39]. However, along with identifying transmissible epigenetic marks, it is vital to understand the role that stable epigenetic regulation has on gene expression. Here we present the first *M. guttatus* methylome. We utilize a novel modeling approach to untangle the complex interactions between methylation and gene expression. We show that non-CG gene body methylation may have a significant effect on gene expression despite occurring at relatively low levels. Utilizing a GO term enrichment approach, we demonstrate that certain functional categories are over-represented in genes with high gene body CG methylation. We provide evidence that there are differences in methylation and gene expression between chromosomes, such that mean gene expression is significantly lower across some chromosomes than others. Finally, we look at transcriptional start sites across the genome, where recent evidence suggests increased recombination in *M. guttatus* [40], and find a corresponding decrease in DNA methylation.

## Methods

### DNA extraction and bisulfite sequencing

We germinated seeds from the *M. guttatus* Iron Mountain inbred line, IM62, the line that was sequenced to establish the *M. guttatus* reference genome [40] ( http://phytozome.jgi.doe.gov). When the second leaf pair of seedlings was just visible we collected leaf tissue from multiple seedlings, flash froze it in liquid nitrogen, and stored it at −80 **°**C. We preformed DNA extractions using a CTAB protocol [41]. We pooled DNA from multiple seedlings before library construction in order to limit the effects of aberrant intra-individual variation [42]. From this pooled sample we generated sequencing template for whole genome bisulfite sequencing (WGBS) following the PBAT (Post-Bisulfite Adaptor Tagging) protocol [43]. With 1 ng of unmethylated lambda DNA obtained from Promega used as a spike-in control for conversion efficiency, 100 ng of genomic DNA from *M. guttatus* was treated with bisulfite using EZ DNA Methylation kit from Zymo Research. Two rounds of random primer extension for tagging bisulfite treated DNA with adaptors were performed using primers for single-end library construction as described in [41]. The concentration of templates was determined by qPCR with Library Quantification Kits from KAPA biosystems. A single lane of 100 cycle reactions on HiSeq 2500 was assigned for the library sequencing.

### Read mapping

We used the software BMap [43] (http://itolab.med.kyushu-u.ac.jp/BMap/index.html) to map bisulifte treated reads to the *M. guttatus* v2.0 reference genome (http://phytozome.jgi.doe.gov). In short, BMap first searches candidate genomic loci for each read in two duplicated genome sequences, one with every C in the genome converted to a T (C2T), and one with G to A (G2A), using an approach called adaptive seed [44]. Next BMap creates pairwise alignments between the read and original DNA sequence of every candidate loci, and calculates scores for each alignment allowing mismatches between T in the reads with C in the reference. Finally an alignment with the highest score is reported for each read. We used default parameters for mapping with BMap. Using alignments exported by BMap, methylation status for every cytosine in every read was called, and counts both supporting the methylated and unmethylated state are assigned for every cytosine residue of the reference genome. Methylation levels for CG, CHG and CHH contexts are exported to different files and analyzed independently.

### Global methylome analysis

We estimated the number of total and methylated cytosines mapped across the genome on a per-nucleotide basis for the *M. guttatus* IM62 seedling methylome. Percent methylation was calculated for each 1 kb window across the genome for total methylation, as well as methylation in each of the three sequence contexts. Centromere positions were estimated from characteristic repeat sequences [45].

### Gene methylation analysis

Using the *M. guttatus* v2.0 annotations [46], we calculated the percent methylation in each sequence context for each of the 24,130 annotated genes. Only the 17,043 for which we had gene expression data [32] were used for down-stream analysis. For each annotated gene we defined three regions: up-stream as the 1kb up-stream of the transcriptional start site, gene body as the transcribed portion of the gene, and down-stream as the 1kb downstream of the 3′ UTR. Gene expression values were generated previously by RNAseq from seedling tissue of genetically distinct *M. guttatus* – a recombinant inbred line derived from cross between divergent populations [32].

In order to determine if gene methylation and expression varied across chromosomes we preformed four ANOVAs with chromosome as an explanatory variable and CG, CHG, CHH, and log-transformed gene expression as response variables.

Gene ontology terms were already assigned to genes [32], and were utilized both to calculate the total number of GO terms per gene, as well as to perform a Fisher’s Exact test to determine what, if any, types of genes were enriched or depleted in our set of highly CG methylated genes, and our set of chromosomes exhibiting significantly reduced gene expression levels.

In order to choose a predictive gene expression model, we included methylation in each of three contexts, percent methylation in gene bodies, up-stream and down-stream regions, intron length (sum of all introns for a gene), exon length (sum of all exons for a gene), number of exons, and interaction terms up to the third degree. Gene length, intron size, and intron number are all known to be positively correlated with gene expression in plants [47], opposite the trend observed in animals [48]. We used a Bayesian information criterion (BIC) [49] to inform our restricted maximum likelihood (REML) model selection (done in order to limit the number of parameters included in our model, and in turn reduce over fitting). Additionally, genes were parsed randomly into thirds, and parameters were tunes for each of these three groups independently. These models were then used to predict gene expression in the remaining to gene groups to provide 3-fold cross-validation [50]. We Z-transformed values to make parameter estimates comparable, making a value of 0 represent the mean value for a variable, with positive or negative deviations reflecting the number of standard deviations a value is from the mean.

We identified transposable elements across the *M. guttatus* genome from the repeat-masked genome assembly [46]. Genomic repeats larger than 100 base pairs were selected and percent methylation in all three sequence contexts was identified for these repeats.

## Results and discussion

### Global methylation

**Fig. 1:**
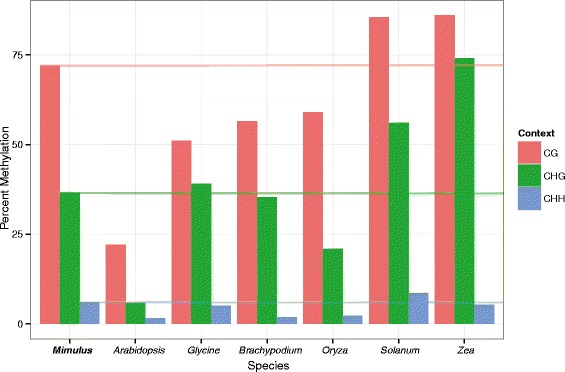
Interspecific comparison of plant DNA methylation levels. A comparison of global DNA methylation levels in CG (red), CHG (green), and CHH (blue) sequence contexts found in *Mimulus guttatus* compared with those of *Arabidopsis thaliana* [66], *Glycine max* [52], *Brachypodium distachyiom* [27], *Oryza sativa* [20], *Solanum lycopersicum* [22], and *Zea mays* [26]

Of the 186 million reads generated, 126 million were mapped to the genome (67.7 % mapping, mean read depth = 19, median = 6). This proportion is typical for *Mimulus* genomic studies eg. [51] given the substantial proportion of the physical genome that is not contained in the v2 reference genome. Mapping to unmethylated lambda DNA confirmed that our PBAT treatment achieved 99.4 % conversion of unmethylated cytosines to thymine. Methylation is most common in a CG context (72 %), intermediate in a CHG context (36.5 %), and lowest in a CHH context (6.1 %) (Fig. 1), with 23 % of total cytosine’s being methylated. The percent of genome methylation found in *M. guttatus* is higher in all contexts than *Oryza sativa* [20], *Arabiopsis thaliana* [8], *Brachypodium distachyiom* [27], lower in all contexts than *Solanum lycopersicum* [22], and both higher or lower than *Zea mays* [26] and *Glycine max* [52] depending on context (Fig. 1). While CHH methylation levels are higher in *M. guttatus* than *Z. mays* and *G. max*, the opposite is true for CHG methylation. CG methylation is highest in *Z. Mays*, moderate in *M. guttatus*, and lowest in *G. max* (Fig. 1).

**Fig. 2:**
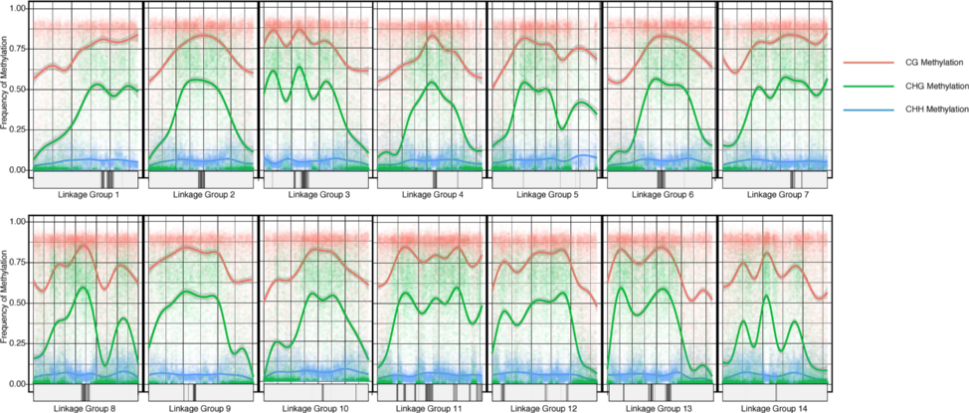
DNA methylation across the *Mimulus guttatus* genome. DNA methylation across the 14 *Mimulus guttatus* linkage groups (putative chromosomes) in all three sequence contexts: CG (red), CHG (green), and CHH (blue). Centromeric repeat densities, adapted from [45], are shown along the X-axis (darker bars indicate higher repeat density). Areas with higher repeat density tend to also have higher DNA methylation. A smoother line [67] was fit across 1kb methylation averages

**Fig. 3:**
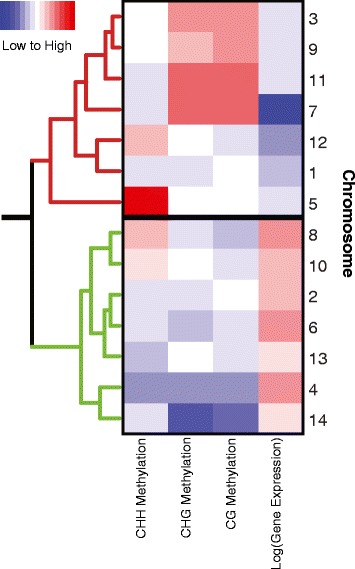
Variation in methylation and expression across chromosomes. A heatmap showing variation in gene expression and methylation across the 14 *Mimulus guttatus* putative chromosomes. The 14 chromosomes clustered into two large groups, those with generally high methylation and low gene expression (top cluster, red dendogram), and those exhibiting the opposite pattern (bottom cluster, green dendogram). On the heat map, red indicates high values and blue indicates low values of methylation or gene expression

Approximate positions of centromeres on *M. guttatus* chromosomes are given by the location and density of centromeric repeats [45]. We confirmed that regions of the genome with high levels of centromeric repeats also tended to have high CG, CHG, and CHH methylation (Fig. 2). We found that gene expression and gene body CG, CHG, CHH methylation varied significantly across chromosomes (log(expression): F_13,17042_ = 4.43, CG: F_13,17042_ = 10.85, CHG: F_13,17042_ = 19.07, CHH: F_13,170423_ = 6.10, p < 0.001)). Chromosomes that have on average higher levels of methylation tended to also have lower gene expression (Fig. 3). From this result, it is unclear whether certain chromosomes are constitutively more highly methylated and transcriptionally silenced, or whether throughout development epigenetic modification at a whole chromosome scale can change the relative expression of genes across entire chromosomes. It does appear that silenced chromosomes have a higher density of heterochromatic repeats, hinting that certain chromosomes may be condensed throughout development.

### Gene methylation

Methylation was significantly depleted in gene bodies relative to both inter-genic regions and transposable elements in all three-sequence contexts (Table 1). While CG methylation was only modestly reduced in gene bodies relative to intergenic regions (Gene Bodies: 56 %, Intergenic: 75 %), CHG (Gene Bodies: 3.8 %, Intergenic: 45 %) and CHH (Gene Bodies: 1.2 %, Intergenic: 7.2 %) methylation levels were drastically reduced (Table 1). Similar results were found in *Oryza sativa* [20], *Arabidopsis thaliana* [53], and *Glycine max* [52]. Methylation both up-stream and down-stream of gene starts was also reduced relative to genome-wide averages. We found that up-stream regions were elevated in non-CG methylation compared to gene bodies, but that up-stream CG methylation was reduced compared to gene body CG methylation (Table 1).

**Table 1:** *Mimulus guttatus* methylation across sequence contexts and genomic regions

	Proportion of cytosines methylated		
	CG	CHG	CHH
Transposable Elements	0.73	0.36	0.063
Gene Body	0.56	0.038	0.012
1^st^ 500bp of Gene Body	0.28	0.032	0.019
Up-stream Regulatory	0.35	0.11	0.027
Inter-Genic Regions	0.75	0.45	0.072
Total	0.72	0.365	0.061

**Fig. 4:**
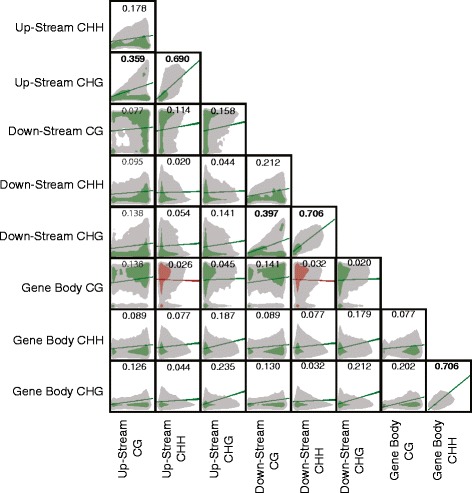
Correlation matrix between forms of methylation at individual genes. Clouds represent density, and lines show the slope of the correlation. Green lines indicate forms of methylation with a positive correlation, while red represents negative correlation. Numbers represent the Pearson correlation (*r*) value, bolded numbers highlight correlations with an r > 0.35. All correlations were found to be statistically significant (n = 17,038, p < 0.05)

The methylation levels in all contexts (CG, CHG, CHH) and genic positions (up-stream, down-stream, and gene body) at a given gene were significantly correlated with one another (Fig. 4). These were positive correlations for all cases but two. The two exceptions were negative correlations between up- and down-stream CHH methylation with gene body CG methylation. The most significant positive correlations were found between CHG and CHH or CG methylation levels at both up-stream and down-stream regions, as well as between CHG and CHH gene body methylation. Interestingly, the methylation levels for all three contexts vary greatly across the three gene regions in a fairly unpredictable manner. For instance, correlation between up-stream CG methylation and gene body CG methylation is only *r* = 0.14. This highlights the disparate functions of regulatory region methylation with that of gene body methylation [54]. The extremely high correlations between CHG and CHH methylation (Fig. 4, *r* > 0.67) in all three regions is likely due to the involvement of similar enzymatic machinery in the propagation of both types of non-CG methylation [55].

### Methylation effect on gene expression

**Fig. 5:**
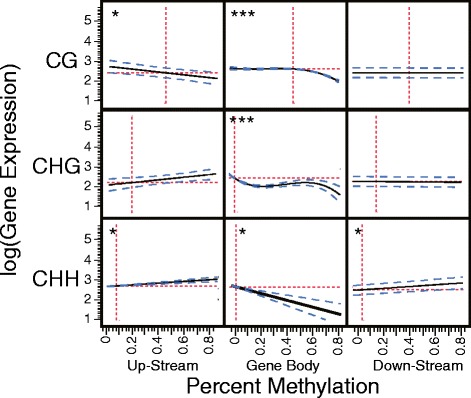
Correlations between DNA methylation and gene expression. A single star represents a significant linear correlation, two stars a significant second-order correlation, and three stars a third order correlation. The red dashed lines represent the means, the black line represents the regression line, and the blue line represents 95 % confidence intervals

A stepwise cubic polynomial model was selected to predict log(gene expression) based on minimum BIC. Out of a possible 454 parameters, the minimum BIC criterion selected a model with 29 factors that explained (R^2^) 20.1 % of the variation in log transformed expression values (*SS* Model: 1764, SS Error: 6981, F_28,17042_ = 153.6, p < 0.0001, Tables 2, 3 and 4, Fig. 5, Additional file 1: Figure S1). Including all 454 parameters increases R^2^ only marginally (to 23.3 %), and the minimum calculated R^2^ calculated in 3-fold cross-validation was 17.9 %. Generally, there is an excess of genes predicted to be expressed at log-transformed values between 1.5 and 2.5, that were actually expressed at levels less than 1.2, as well as genes expressed above 4, which this model never predicts (Additional file 1: Figure S1). It is clear that while gene methylation can modify gene expression, it cannot predict the complete repression, or extremely high expression of some genes. As all parameters were Z-transformed prior to modeling, the effect estimates are comparable across variables (Table 4). In order to maintain both statistical and molecular consistency throughout, both Z-transformed values and raw values are reported. The inclusion of both various forms of DNA methylation and gene architecture (number of exons, exon length, intron length) have not been included in a single model explicitly testing their ability to predict gene expression, but their independent effects have often been looked at in relation to gene expression. While it is hard to compare our integrative analysis on gene expression with prior studies, we generally find the same direction of effect in our data as was found in other plant systems [3]. Trends are thus not *Mimulus* specific, but likely more general effects of DNA methylation on gene expression in angiosperms. Finally, when discussing the role of various forms of methylation on gene expression we often designate a specific type of methylation as having a positive or negative effect on gene expression. In this context that indicates that there was significant predictive ability for a given type of methylation on gene expression. However, due to the nature of this experimental design we cannot definitively define the arrow of causation.

**Table 2:** Summary of REML genetic architecture and methylation fit on log transformed gene expression

R-Square	0.201775
R-Square Adj.	0.200462
Root Mean Square Error	0.640578
Mean of Response	2.483507

**Table 3:** Analysis of variance in gene expression predictive model

Source	DF	Sum of Squares	Mean Square	F Ratio
Model	28	1764.7903	63.0282	153.6
Error	17014	6981.5210	0.4070	Prob > F
C. Total	17042	8746.3113		p < 0.0001

**Table 4:** Sorted estimate of parameter effects on log transformed gene expression

Positive Terms	Estimate	Std Error	t Ratio	Prob > |t|
Intron Length	0.3472	0.0117	29.75	<.0001
Gene Body CHG^2^	0.0874	0.0082	10.64	<.0001
Number of Exons*Intron Length	0.0793	0.0081	9.74	<.0001
Exon Length	0.0767	0.0102	7.55	<.0001
Exon Length* Intron Length	0.0553	0.0070	7.86	<.0001
Gene Body CG * Exon Length	0.0392	0.0089	4.4	<.0001
Gene Body CG^2^ * Exon Length	0.0303	0.0069	4.37	<.0001
Up-Stream CHH	0.0275	0.0055	4.99	<.0001
Gene Body CG*Gene Body CHH	0.0244	0.0064	3.78	0.0002
Down-stream CHH	0.0185	0.0050	3.69	0.0002
Up-stream CHH* Percent CG	0.0167	0.0051	3.28	0.0011
Intron Length^3^	0.0105	0.0007	14.4	<.0001
Exon Length^2^ * Number of Exons	0.0074	0.0009	8.19	<.0001
Negative Terms				
Gene Body CHG	−0.3273	0.0197	−16.58	<.0001
Intron Length^2^	−0.1611	0.0076	−21.18	<.0001
Gene Body CG^2^	−0.0980	0.0092	−10.62	<.0001
Gene Body CG	−0.0720	0.0118	−6.09	<.0001
Exon Length * Number of Exons	−0.0662	0.0076	−8.72	<.0001
Gene Body CHH	−0.0451	0.0076	−5.93	<.0001
Number of Exons	−0.0308	0.0112	−2.75	0.0059
Percent CG^3^	−0.0277	0.0059	−4.73	<.0001
Up-Stream CG	−0.0274	0.0054	−5.06	<.0001
Exon Length^2^	−0.0205	0.0033	−6.28	<.0001
Gene Body CHG * Exon Length	−0.0198	0.0058	−3.41	0.0007
Up-stream CG* Up-stream CHH	−0.0188	0.0058	−3.23	0.0012
Up-stream CG* Gene Body CG	−0.0170	0.0052	−3.28	0.001
Exon Length * Intron Length * Number of Exons	−0.0118	0.0016	−7.21	<.0001
Gene Body CHG^3^	−0.0063	0.0008	−7.95	<.0001

*Superscripts represent the power to which a term is raised

#### Gene body CG methylation

**Fig. 6:**
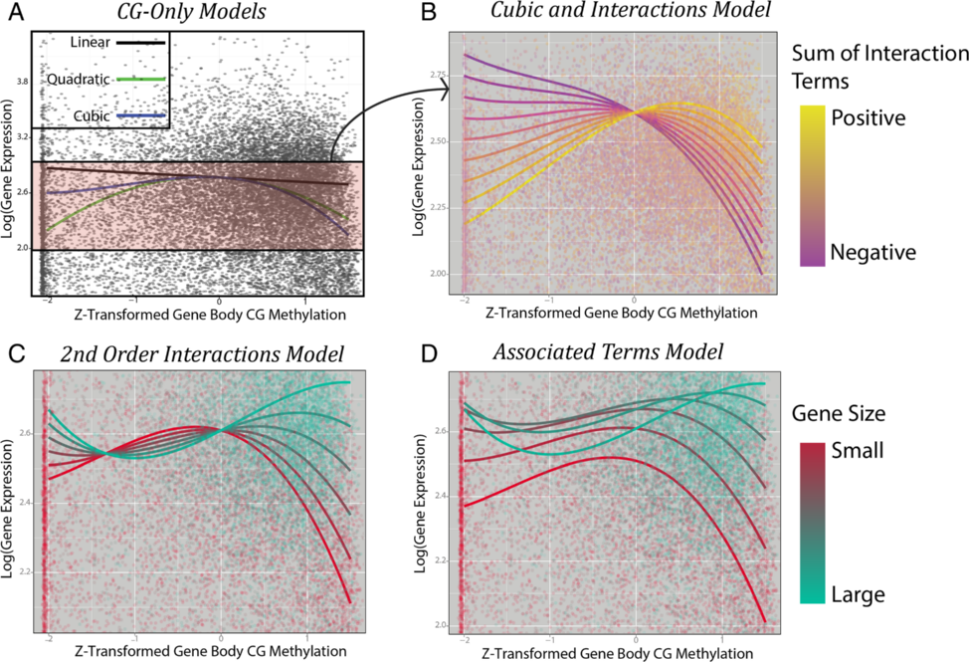
DNA methylation modeling to predict gene expression. A visual depiction of our simplified model showing the effect of gene body CG methylation and an increasing complexity of interaction terms on gene expression. **a** A scatterplot comparing Z-transformed gene body CG methylation values with log(gene expression) values. The black line shows the linear term, green line includes both the linear and quiadratic term, and the blue line includes linear, quadratic, and cubic terms. **b** Interaction plot depicting the interaction between gene CG methylation and exonlength, up-stream CHH methylation, gene body CHH methylation, and gene body CG methylation on gene expression. Summed terms across these four terms are considered ranging from −1.6 (dark purple) to 1.6 (yellow). Points represent actual genes CG gene body methylation, gene expression, and their color represents their interaction sum on the same scale as the model colors. **c** The second order interaction term of squared gene body CG methylation by exon length is added to the model depicted in **b**. As exon length increases (goes from red to blue) gene body CG methylation is found to have a more positive effect on gene expression. Points represent genes, and colors represent the exon length of these genes on the same scale as the model colors. **d** The independent effect of exon length on gene expression is added to the model depicted in **c**. The shape of the lines does not change, however predicted gene expression is altered (the lines move up or down on the y-axis) depending on the predicted effects of exon length on gene expression

*Linear Effects:* log(***GE***) = 2.61 − 0.07***m***_***cg***_ = ***f***^  1^, where m_cg_ is gene body CG methylation and GE is gene expression. Controlling for gene architecture and other forms of methylation, we observe a negative linear effect of gene body CG methylation on gene expression (Figs. 5 and 6a. black line). The effect size of gene body CG methylation (*m*_*cg*_) is −0.07 (Table 3); a gene with *m*_*cg*_ = − 1 (32 %) is predicted to have 35 % higher expression than one with *m*_*cg*_ = 1 (80 %) (Fig. 6a, black line). Previous studies report that gene body CG methylation is positively correlated with gene expression [3, 10, 19, 20]. While the linear component of the model seems to contradict these previous reports, it cannot be interpreted in isolation. The polynomial and interaction terms indicate that gene body methylation has neither universally positive nor negative effects on gene expression. Traditional methods that looked for associations between gene expression and gene body CG methylation (which find a positive correlation between the two), and modeling methods as applied here followed by only analysis of the simple linear terms (which finds a negative correlation) come up quite short in portraying the role of gene body CG methylation in transcriptional regulation. By considering non-linear effects of methylation on gene expression we can begin to increase our understanding of the role of gene body CG methylation in gene regulation.

*Quadratic Effects*: ***f ***^1^ − 0.1***m***_***cg***_^2^ = ***f ***^2^. The squared gene body CG methylation term has the second largest effect size of any methylation term (after gene body CHG methylation) on gene expression, and leads to a predicted local *m*_*cg*_ maximum for gene expression (due to it being a negative parabola, Fig. 6a, green line). This maximum is found at *m*_*cg*_ = − 0.35 (47 %). As gene methylation increases or decreases relative to a moderate 45 % methylation, gene expression is expected to decrease (Fig. 6a; green line).

*Cubic Effects:****f ***^2^ − 0.03***m***_***cg***_^3^ = ***f ***^3^. The cubed gene body CG methylation term is also negative; compared to our quadratic model, this leads to higher predicted gene expression for genes with lower than average methylation, and lower for genes with higher than average methylation. This slightly lowers the predicted local maximum of gene expression to *m*_*cg*_ = − 0.43 (45 %) (Fig. 6a, blue line). These data agree with previous findings that there is a non-linear relationship between gene body CG methylation and gene expression with an intermediate optimum [3].

#### Interaction terms

*Negative Promoter CG Methylation Interaction:****f ***^3^−.02***m***_***cg***_***u***_***cg***_ = ***f ***^4^. The effect of interaction terms in this model is best understood by comparing expected gene expression across *m*_*cg*_ values for a variety of interaction term values. Changes in linear interaction term values (in this case up-stream cg methylation *u*_*cg*_), lead to a change in our linear *m*_*cg*_ coefficient. For example, at *u*_*cg*_ = 1 (82 %), 0.2*m*_*cg*_ is subtracted from our earlier model, we are left with:

$$ \log (GE)=2.61-.07{\boldsymbol{m}}_{\boldsymbol{cg}}-.10{m}_{cg}^2-0.03{m}_{cg}^3-.02{\boldsymbol{m}}_{\boldsymbol{cg}}={f}_{u=1}^3 $$

$$ {f}_{u=1}^3=2.61-.09{\boldsymbol{m}}_{\boldsymbol{cg}}-.10{m}_{cg}^2-0.03{m}_{cg}^3 $$

At *u*_*cg*_ = 1 (82 %) we find that the local maximum for gene expression is at *m*_*cg*_ = − 0.62 (40.2 %), while at *u*_*cg*_ = − 1 (24.1 %) the local maximum for gene expression is at *m*_*cg*_ = − 0.29 (48.8 %). As up-stream CG methylation decreases (Fig. 6b, from purple to yellow lines), gene body CG methylation is expected to have a more positive effect on gene expression.

While it has long been noted that regulatory region methylation is linked with reduced gene expression, here we find evidence that the difference in methylation between these regions also appears to correlate with gene expression. The negative interaction term between up-stream and gene body CG methylation predicts that distinctly different levels of methylation up-stream and within genes tends to correspond with higher levels of gene expression. When gene body CG methylation and regulatory methylation are both high, gene expression tends to be low (Fig. 6b, purple lines at high gene body CG values). However, as either decreases (Fig. 6b, purple lines at low gene body CG values, or yellow lines at high CG methylation values), gene expression is expected to increase.

*Three positive interaction terms:****f ***^4^ + ***m***_***cg***_(.02***u***_***chh***_+.02***m***_***chh***_+.04***l***_***exon***_) = ***f ***^5^: While only up-stream CG methylation showed a negative interaction with gene body CG methylation, three terms have positive linear interactions: Up-stream CHH methylation, gene body CHH methylation, and exon length. These can be treated in much the same way as our negative interaction term. Depending on the values of these terms, they can offset each other and lead to the removal of any interaction effect. For example if exon length (*l*_*exon*_) = − 1 and up-stream (*u*_*chh*_) and gene body (*m*_*chh*_) CHH methylation = 1 these positive interaction terms cancel out (−.4 + .2 + .2 = 0). However, if we consider them varying in the same direction, they can have a striking effect on the relationship between gene body CG methylation and gene expression. At *u*_*chh*_ = *m*_*chh*_ = *l*_*exon*_ = 1 (and the negative interaction term *u*_*cg*_ = 0), we see the local maximum is at a methylation level of *m*_*cg*_ = − 0.05 (55.1 %). If our negative interaction term *u*_*cg*_ = − 1 , this increases to *m*_*cg*_ = + 0.05 (57.3 %) (Fig. 6b, varying the values of our interaction terms, *u*_*chh*_ = *m*_*chh*_ = *l*_*exon*_ = − *u*_*cg*_ from −1.6 to 1.6, as the summed interaction term increases (lines become yellow) the local maxima for gene expression does so as well). When *u*_*chh*_ = *m*_*chh*_ = *l*_*exon*_ = − *u*_*cg*_ < 0, gene body CG methylation is almost purely repressive. At a summed interaction value less than −0.7 there is no longer a local maximum, and CG methylation has a purely negative effect on gene expression.

*Quadratic Interaction Terms:* log(***GE***) = ***f ***^5^+.03***m***_***cg***_^2^***l***_***exon***_ = ***f ***^6^. Finally the interaction between the quadratic gene body CG methylation term and exon length is included in this model. As our quadratic term increases, not only does the position of the local gene expression maximum increase, so to does the inflection point (the point at which the function changes from concave to convex). Now, at the same linear interaction values tested above (*u*_*chh*_ = *m*_*chh*_ = *l*_*exon*_ = − *u*_*cg*_ = 1), our local maximum occurs at *m*_*cg*_ = 0.07 (57.8 %) (Fig. 6c). As exon sizes increase, the effect of gene body CG methylation is expected to rapidly become more positive, and peak gene expression is predicted to occur at higher *m*_*cg*_ levels. At *l*_*exon*_ = 3 (3.5 kb), we find that the local maximum for gene expression occurs at *m*_*cg*_ = 0.90 (78.1 %) and at *l*_*exon*_ = 4 (6kb) there is no longer a local maximum for *m*_*cg*_, and the highest expected gene expression occurs at *m*_*cg*_ approaching 100 % (largest gene size in Fig. 6c). It appears that for genes with smaller exons, moderately methylated genes are most highly expressed, but as genes become larger so to does the level of gene methylation that is associated with more highly expressed genes. Our gene size by gene body CG methylation results confirm a pattern observed by Zilberman et al. [3] in the first genome-wide methylome analysis in *Arabidopsis* in which found only a marginal relationship between gene size and gene expression, except for the genes in which gene bodies were methylated and then they found a positive relationship between gene size and gene expression.

*Individual effects of interaction terms:* log(***GE***) = ***f ***^5^ + .08***l***_***exon***_ − .02***l***_***exon***_^2^ = ***f ***^6^: Finally, we consider the effect of multiple terms simultaneously. Up until this point we have only included gene body CG methylation effects, and its interaction terms, while not including the independent effects of the term with which it interacts. Independent of gene body CG methylation, we find that gene expression tends to increase as the standardized exon length increases from −1 (500bp) to 2 (2kb), and beyond this point we expect a decline. In the absence of interaction terms, only considering independent effects of gene body CG methylation and exon length, we would estimate that peak gene expression occurs at an exon length of 2kb, and methylation of 45 %. Here we show that the effect of gene body CG methylation on expression is extremely size dependent, and that gene expression is expected to be highest for large highly gene body CG methylated genes, but lowest for small highly gene body CG methylated genes (Fig. 6d). It may be that as exon length increases, gene methylation is necessary to stabilize transcription, while for smaller genes it is not necessary for this purpose, and rather plays a repressive effect due to condensing chromatin near the transcription start site.

In this same way all other independent and interaction terms could be added to this model, parameters considered, and hypotheses tested. As nine distinct parameters are included (with 27 total terms) in this model the results quickly become difficult to conceptualize or visualize, yet through full-model construction, followed by simplification methods as presented above it is possible to decipher complex higher order regulatory interactions. We briefly discuss the effects of the other significant gene size and methylation terms in this model.

#### Intron length

Intron length shows significant first, second, and third order effects with a gene expression peak at an intron size of approximately 1700 base pairs. Additionally, a positive interaction term with both exon length and number of introns suggests that generally, longer genes with more introns tend to be more highly expressed. Although relatively large genes do tend to be most highly expressed, there are negative quadratic terms for both exon and intron length that suggest after a certain point, increasing exon and intron length should be associated with decreased gene expression.

#### Non-CG gene body methylation

Gene body CHG methylation had significant linear, quadratic, and cubic independent terms, and an exon length interaction term. Gene body CHG methylation has a negative effect on gene expression across nearly its full range of possible values (Fig. 5), and it appears that it is the increase from no CHG methylation to slight CHG methylation that reduces gene expression. After this point the effect of CHG methylation appears to be minimal. The negative exon length interaction term suggests that long genes with CHG methylation tend to be more significantly repressed than smaller genes.

Gene body CHH methylation was found to have a negative effect on gene expression (Fig. 5), but a positive interaction with gene body CG methylation. Thus, as gene body CHH methylation increases, gene body CG methylation is expected to have a more positive effect on gene expression, but mean gene expression, independent of gene body CG methylation, is expected to decrease. Like CHG methylation, a manual inspection reveals that the jump from no CHH methylation to low levels of CHH methylation leads to a decrease in gene expression, but after this, the effects of increased methylation are minimal.

While it has been suggested that non-CG gene body methylation may be misattributed to genomic regions that are actually pseudogenes or paralogs [52, 56], here we find evidence that in at least some cases these genes are still expressed, albeit at lower levels than non-methylated genes. One possible explanation is that non-CG methylation of genes may be a first step on the path toward pseudogenization [57], whereby genes become targeted by non-CG methylation, gene expression is reduced, mutational constraints become lightened, and eventually the gene becomes entirely non-functional. Additionally, it may be that tightly developmentally controlled small RNAs are responsible for the majority of this methylation, and the use of identical tissue for methylation and gene expression analysis would identify a stronger role of gene body non-CG methylation on gene expression. Finally, even trace amounts of non-CG gene body methylation may be indicative of the presence of small RNAs, and RNA-directed DNA methylation (RdDM) [58]. It could be that the methylation of just a few nucleotides by a single 24nt siRNA is enough to reduce gene expression, without significantly altering the methylation state of the whole gene.

#### Regulatory region methylation

Along with a negative interaction with gene body CG methylation, up-stream CG methylation also has a direct negative effect on gene expression (Fig. 5) and a negative interaction with up-stream CHH methylation. Not only does up-stream CG methylation limit the positive effect of gene body CG methylation on predicted gene expression, it also directly reduces predicted expression. Up-stream CHH methylation has both a significant positive linear effect on gene expression (Fig. 5), and a positive interaction with gene body CG methylation. The negative interaction term with up-stream CG methylation suggests that while up-stream CHH methylation generally has a positive effect on gene expression, when it is found alongside CG methylation, this effect is negated. While down-stream CHH methylation did not interact with gene body CG methylation, it was also found to have a positive effect on gene expression (Fig. 5).

A previous study in *Arabidopsis* similarly found that there was a positive correlation between gene expression and regulatory CHH methylation (albeit not in a regression framework) [26]. They posit that as gene expression increases, unstable transcripts are produced as by-products at both the 5′ and 3′ ends of genes. In turn, this lead to the production of small RNAs that can target and cause CHH methylation bracketing highly expressed genes through RNA directed DNA methylation (RdDM). The possibility that increased gene expression causes increased regulatory CHH methylation, and not vice-versa does not introduce bias in this framework, but rather reinforces that our interpretations do not imply causality.

#### Gene expression modeling overview

While the traditional method of looking for simple associations between methylation state and gene expression has provided some insight into epigenetic regulation, here we demonstrate that modeling approaches can provide additional insight into these systems. We explain a surprisingly high (20.1 %) amount of the variation in log(gene expression) simply through methylation and gene architecture variation. We considered a potential 454 parameters in our model before settling on 29, but it is important to note that many other factors such as presence of enhancers within the gene body and distance to transposable elements, likely also modify the role of methylation on expression. By considering exon and intron length within this model we take the first steps to account for these potential confounding factors of methylation on expression. It is worth stressing that the gene expression and methylome data were not only collected from different individuals, but also different genetic lines, using different vegetative tissue types, and grown under slightly different greenhouse conditions. It is certainly possible that a similar model, tuned across multiple paired methylome and gene expression samples, could predict gene expression with greater precision. This portion of gene expression variation explained represents that which is at least relatively stable across individual genotypes, tissue, and conditions. While here we apply this model to gene expression on a gene-by-gene basis, through altering the response variable to another parameter of a gene, such as it’s mutation rate, gene expression variance, or the tissues in which it is expressed, this model could be extended to look for other roles of DNA methylation on gene function and evolution.

Results from this and other [3, 20, 29, 59] studies suggest that gene body CG methylation needs to be considered to have a quadratic effect on gene expression, and that this effect is highly dependent on exon size. Thus, genes can either be parsed according to exon length prior to estimating the role of gene body CG methylation on expression, or the interaction between exon length and methylation should be considered in the model. Other forms of methylation appear to have a more straightforward role in regulating gene expression, and in some cases it may suffice to predict that, for example, as up-stream CG methylation increases at a gene, its expression will likely decrease.

### Gene ontology analysis of genes with high CG gene body methylation

**Fig. 7:**
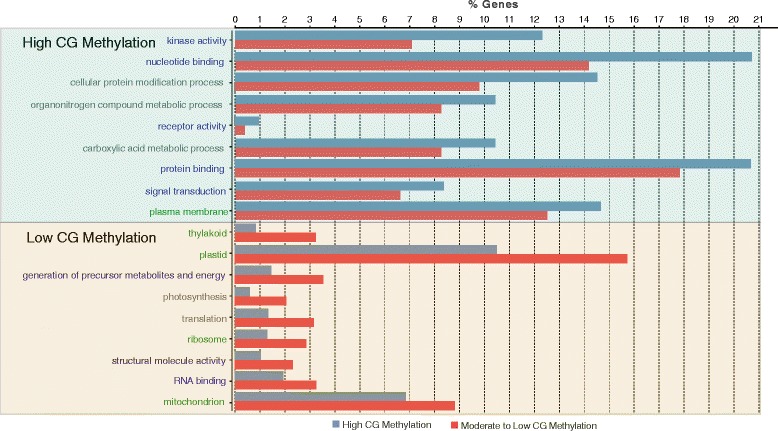
Gene Ontology classes over and underrepresented in highly gene body methylated genes. Genes Ontology terms significantly enriched and depleted in genes in the top 10 % for gene body CG methylation. X-axis shows the percent of genes in both the high CG methylated portion (blue) as well as the remainder of the transcriptome (red) that contained the given GO terms. Text color represents the class of GO term: blue-molecular functions, grey-biological processes, and green-cellular component

Comparing genes in the top 10 % genome wide for gene body CG methylation with the remainder of the genome, we found numerous gene categories that are either enriched or depleted in our set of highly CG methylated genes. Genes coding for proteins with kinase activity, involved in signal transduction, and nucleotide binding were among those which tended to be highly methylated, while proteins functioning in the thylakoid, plastid, and ribosome, as well as proteins involved in primary metabolism, photosynthesis, and RNA binding tended to be lowly or moderately methylated (Fig. 7). Similar results have been found in *Brachypodium*, rice [29], and *Arabidopsis* [3].

### Decreased methylation near transcription start sites

**Fig. 8:**
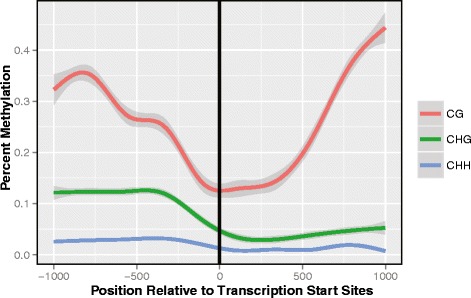
DNA methylation around transcriptional start sites. Around gene start sites, and persisting into the first 500 base pairs of the gene body, we observe a significant drop in DNA methylation. For CG (p-value = 6.45 × 10^−55^), CHG (p = 3.38 × 10^−100^), and CHH (p = 4.61 × 10^−308^) methylation was significantly reduced in the first 500bp of the gene relative to the up-stream regions. Both CG (p = 3.55 × 10^−138^) and CHG (p = 4.04 × 10^−11^) methylation then significantly increases over the next 500 bp, while for CHH it continued to decline (p = 8.93 × 10^−10^)

We looked for changes in methylation near gene transcription start sites. We found that CG, CHG, and CHH methylation all were significantly depleted at and around gene start sites (Fig. 8). This depletion, along with the negative interaction term between up-stream and gene body CG methylation on gene expression, points towards a role of methylation in epigenetically labeling coding genetic regions. Additionally, recent evidence has shown that in *M. guttatus* genetic recombination occurs at higher frequency near gene start sites. In other systems it has been shown that DNA methylation is negatively correlated with recombination [7], and it may be that decreased methylation at gene start sites is related to the increase in recombination.

Decreased methylation near transcription start sites (TSS) was one of the earliest discovered phenomena of gene methylation [3]. However, new evidence in *M. guttatus* [40] provides us with a novel framework in which to view this pattern. Hellsten et al. [40] identified an approximately two-fold increase in recombination near gene start sites (the beginning of exon 1 being most enriched), and postulated that this may be related to nucleosome depleted open chromatin at these regions as is the case in *Arabidopsis* [60] and rice [61]. At the time of their publication however, there was no evidence for a similar trend in *Mimulus*. Here, evidence of depleted methylation near TSS (Table 1; Fig. 8) provides support to the theory that open chromatin (unmethylated) near TSS may increase local recombination rates. It appears that at least in yeast double stranded breaks occur most frequently in open chromatin regions [62], which may explain the observed increase in recombination near transcription start sites. It is likely that the increased recombination near TSS is simply a by-product of the dual forces exerted by DNA methylation, one involved in gene regulation, and another limiting double stranded breaks. The ability for DNA methylation to alter both of these processes provides an interesting link between gene regulation and DNA recombination that may or may not prove to be of evolutionary significance. Further studies linking methylation and recombination at a nucleotide level should further clarify this trend.

### Transposable element methylation

We identified 1,411 transposable elements across the genome ranging in copy number from 1 to 2,380 (median copy number = 7). Percent methylation was calculated in each of three sequence contexts. In total, 34 % of the *M. guttatus* genome was estimated to be of transposable element sequence, and methylation levels within transposable elements were significantly higher than that of genes, and at similar levels to inter-genic regions (Table 1). We did not find there to be a significant copy number effect on TE methylation. Of the top 25 most common transposable elements in the *Mimulus* genome, six were type 1, and 19 were type 2 transposons (Table 5).

**Table 5:** Transposable element frequencies, classes, and methylation

		Percent Methylation				
ID	Copies	CG	CHG	CHH	Family	Class
helB8c	2380	0.809	0.473	0.052	Helitron	2
MULE_MITE1c	674	0.627	0.263	0.102	MITE	2
Copia1b	424	0.780	0.437	0.058	Copia	1
helD8b	402	0.712	0.357	0.055	Helitron	2
MULE_MITE2b	245	0.633	0.285	0.077	MULE	2
pogo_MITE2b	203	0.738	0.281	0.071	MITE	2
MULE_MITE16b	200	0.713	0.207	0.070	MULE	2
hAT_MITE1	197	0.782	0.294	0.051	MITE	2
MULE_na62	165	0.768	0.359	0.064	MULE	2
MULE_MITE1a	158	0.720	0.250	0.071	MULE	2
LARD4	155	0.793	0.442	0.081	LARD	1
hAT_na66a	151	0.869	0.276	0.042	hAT	2
Tourist6c	151	0.634	0.259	0.071	MITE	2
MuDR8	150	0.791	0.492	0.089	MuDR	2
MULE_na13a	145	0.752	0.400	0.068	MULE	2
Copia1a	143	0.717	0.374	0.045	Copia	1
Copia2	137	0.685	0.494	0.085	Copia	1
SINE1a	134	0.685	0.293	0.112	SINE	1
Gypsy8	128	0.605	0.228	0.033	Gypsy	1
MULE_na13b	128	0.449	0.260	0.058	MULE	2
helF3c	119	0.737	0.362	0.067	Helitron	2
Jittery7	116	0.639	0.260	0.053	Mu	2
Toursit4c	115	0.781	0.315	0.085	MITE	2
Gypsy4	111	0.818	0.402	0.051	Gypsy	1
MULE_MITE25b	109	0.626	0.151	0.042	MULE	2

We find that DNA methylation in all contexts is enriched in transposable elements relative to genes, however this is most significant for non-CG methylation (Table 1). This suggests that both RNA dependent DNA methylation (RdDM) is targeting and silencing transposable elements in *M. guttatus* as is this case in other angiosperms. Found at 2,380 copies, the helB8c family of helitron elements is far and away the most common transposon in the *Mimulus* genome (more abundant than the next seven TE families combined; Table 5). Helitrons are a relatively newly discovered class of type 2 transposable elements that propagate through a rolling circle mechanism that is still somewhat mysterious [63]. One thing that is clear, is that these elements have been highly successful in propagating across flowering plants, making up 2 % of the *Arabidopsis* genome [64]; a single family of helitrons makes up 6 % of the maize genome [63], making it the most abundant DNA transposon identified. Here, we provide evidence for the success of these elements across the diversity of flowering plants.

## Conclusions

Much remains unknown about the gene regulatory information contained in an organism’s methylome, but here we provide further evidence of complex interactions between gene methylation and expression. DNA methylation may actively alter gene expression, itself be altered by gene expression, or both methylation and expression may be jointly determined by a distinct genetic feature. Still the ability to explain over a fifth of the variation in log transformed gene expression by local DNA methylation, and basic genetic architecture (exon length, intron length, exon number), is promising and has numerous potential applications. Recent efforts have shown that the plant methylome is relatively stable throughout development [65], unlike gene expression. In this way methylation at a gene likely reflects moderately stable epigenetic control of gene expression, while developmentally activated transcription factors and small RNAs may provide highly plastic gene expression control throughout development. Through combining differential methylation analyses across tissue types, environmental treatments, or genetic lines with a modeling approach as described here; our understanding of the role of epigenetic variation in gene regulation can be greatly increased.

## Additional file

Additional file 1: Figure S1. Predicted log (gene expression) from cubic polynominal REML model compared to actual log (gene expression). Slope =1.02, R^2^ = 0.201, df = 28.
